# Attachment Security Balances Perspectives: Effects of Security Priming on Highly Optimistic and Pessimistic Explanatory Styles

**DOI:** 10.3389/fpsyg.2016.01269

**Published:** 2016-08-25

**Authors:** Yanhe Deng, Mengge Yan, Henry Chen, Xin Sun, Peng Zhang, Xianglong Zeng, Xiangping Liu, Yue Lye

**Affiliations:** ^1^Beijing Key Laboratory of Applied Experimental Psychology, School of Psychology, Beijing Normal UniversityBeijing, China; ^2^The International Focusing Institute, NyackNY, USA; ^3^Department of Psychology, The Chinese University of Hong KongHong Kong, China

**Keywords:** optimistic explanatory style, pessimistic explanatory style, attachment security, security priming, self-serving attribution, self-deprecating attribution, self-enhancing defense

## Abstract

Highly optimistic explanatory style (HOES) and highly pessimistic explanatory style (HPES) are two maladaptive ways to explain the world and may have roots in attachment insecurity. The current study aims to explore the effects of security priming – activating supportive representations of attachment security – on ameliorating these maladaptive explanatory styles. 57 participants with HOES and 57 participants with HPES were randomized into security priming and control conditions. Their scores of overall optimistic attribution were measured before and after priming. Security priming had a moderating effect: the security primed HOES group exhibited lower optimistic attribution, while the security primed HPES group evinced higher scores of optimistic attribution. Furthermore, the security primed HOES group attributed positive outcomes more externally, while the security primed HPES group attributed successful results more internally. The results support the application of security priming interventions on maladaptive explanatory styles. Its potential mechanism and directions for future study are also discussed.

## Introduction

Based on the model of learned helplessness (Abramson et al., 1978), Seligman et al. (1979) defined the concept of explanatory style as individuals’ habitual explanations toward events of success and failure. The construct of explanatory style consists of three dimensions: internal-external, global-specific and stable-unstable (Peterson and Seligman, 1984). According to an individual’s scores on these three dimensions, explanatory style is broadly divided into optimistic or pessimistic explanatory styles (Zullow et al., 1988). Individuals with an optimistic explanatory style tend to make internal, stable, and global attributions following successful events (e.g., I got an A in the final exam because of my higher intelligence), and the opposite when failures occur (e.g., I failed in the final exam because the room was noisy). In contrast, a person with a pessimistic explanatory style is likely to generate such internal, stable, and global explanations for failures (e.g., I failed because of my lower intelligence) and the opposite for success (e.g., I got an A because the test items were easy; Gillham et al., 2001).

Numerous studies have shown that an optimistic explanatory style not only positively affects psychological and physical performance in humans (Hirsch et al., 2009; Borsuk, 2013), but also positively predicts successful careers (Boyer, 2009; Tsai, 2010, unpublished). However, other studies have found that optimistic explanatory style does not always contribute to healthy psychological functioning or career success, and may even have maladaptive effects. First, as suggested by Coyne and Tennen (2010), the benefits of optimistic explanatory style seem to be inconsistent. For example, an online survey study showed that optimistic explanatory style had no significant relationship with doctoral program retention (Richards, 2012). Another study found that optimistic explanatory style had a weak predictive value for GPA among undergraduates (Maleva et al., 2014). Second, an optimistic explanatory style may have a negative impact in certain contexts. For example, optimistic explanatory style was negatively correlated with the immune status of HIV-infected men, as measured by helper-inducer lymphocytes (CD4), both cross-sectionally and prospectively (Tomakowsky et al., 2001). Similarly, as a prospective study of healthy women showed, dispositional optimists showed more immune system decrements following a persistent stressor (Cohen et al., 1999). Moreover, as Isaacowitz and Seligman (2001) found, elderly adults with an optimistic explanatory style were at a higher risk for depressive symptoms when faced with negative life events.

We think that these mixed findings relate to the degree of one’s optimistic explanatory style, i.e., while a moderately optimistic explanatory style may be adaptive, an exaggerated optimistic explanatory style may be maladaptive. First, it has been demonstrated that optimistic explanatory style, as measured by the Attributional Style Questionnaire (ASQ; Peterson et al., 1982), is correlated with self-serving attributional bias (Jari-Erik, 1992; Lee and Seligman, 1997). As suggested by Alloy et al. (1990) and Taylor and Brown (1994), excessively unrealistic self-serving attributional bias may result in illusions of invulnerability. Thus, highly optimistic explanatory style (HOES) represents an exaggerated self-serving attributional pattern, which, due to inadequately perceiving risk, delays dilemma coping and undermines performance (Izawa, 2011). Moreover, self-serving attributional bias and optimistic explanatory style are both defined by internal attributions of success and external attributions of failure (Bradley, 1977; Campbell and Sedikides, 1999). Hence, individuals with HOES are more likely than the moderately optimistic to exhibit unrealistic illusions in order to maintain positive beliefs about the self. In sum, HOES, rather than optimistic explanatory style in general, may act as a self-enhancing defense that ignores negative outcomes and exaggerates positive ones, potentially compromising psychological health over the long term (Robins and Beer, 2001).

On the other hand, research has consistently shown that pessimistic explanatory style correlates more simply with psychological and physical dysfunction, as well as career failure (Dykema et al., 1995; Peterson and Vaidya, 2001; Levy et al., 2009). These negative effects of a highly pessimistic explanatory style (HPES) may well be understood from the perspective of learned helplessness (Peterson and Park, 1998). Contrary to those with HOES, individuals with HPES tend to respond to negative events by appraising themselves more negatively, and making more self-blaming attributions.

Although HOES is quite different from HPES, attachment insecurity may underlie both of these dysfunctional attributional patterns. Attachment styles – the stable patterns of interpersonal behaviors, emotions, and expectations derived from particular histories with early caregivers – are divided into secure and insecure attachment styles (e.g., Mikulincer and Shaver, 2007). Secure attachment results from repeated experiences with responsive and consistent caregivers, and is characterized by trusting in one’s own worth and in the support of others in times of need. Insecure attachment, on the other hand, is conceptualized in terms of attachment avoidance and attachment anxiety (Brennan et al., 1998). Attachment avoidance results from repeated experiences with unavailable or non-responsive caregivers; such individuals tend to distrust others and emphasize their own strength and autonomy. In times of need, an avoidant person uses deactivating strategies to deny their dependence on others, and one such strategy is to adopt a defensively optimistic attributional style. Meanwhile, attachment anxiety results from repeated experiences with inconsistent caregivers, and leads to an ever-vigilant worry about being abandoned. These individuals rely on hyperactivating strategies to cope with threats of loss. A pessimistic explanatory style that seeks to evoke others’ closeness and support is one such strategy.

Several studies have associated attachment insecurity with these maladaptive attributional patterns. Avoidant-attached individuals tend to show a defensively self-serving attributional pattern. Mikulincer (1995) found that avoidant individuals reacted to self-threatening information by defensively inflating self-attributes. These individuals tend to attribute positive outcomes to more internal, stable, global, and controllable causes, and the reverse for negative outcomes (Man and Hamid, 1998), such as avoidant-attached students who blamed others for their failed test (Kogot, 2002, unpublished). Further, avoidant individuals were found to flatter themselves by deprecating others’ motivation, such as cynically explaining their partner’s positive behavior (Collins et al., 2006). While these strategies may seem to be self-serving, they become inhibited under cognitive load (Mikulincer, 1998), rendering avoidant individuals without adequate internal resources to cope.

Anxious attachment, on the other hand, has been associated with a pessimistic attributional pattern. For example, anxious-attached adults are more likely to attribute threat-related events to uncontrollable causes and global personal inadequacies (Shaver et al., 2008). These individuals are also more likely to exaggerate the threatening aspect of events and to doubt their ability to deal with the threat (Mikulincer et al., 2003). This association has been found in youth as well. A study of children found a positive correlation between attachment insecurity and pessimistic attributional style (Goldner et al., 2015). Also, a study of early adolescents showed that pessimistic attribution of negative events fully mediated the correlation between anxious attachment and depressive symptoms (Kamkar et al., 2012).

Since attachment insecurity, whether anxious or avoidant, may be a risk factor for HOES as well as HPES, we propose that enhancing one’s sense of attachment security may attenuate these patterns. Previous studies have demonstrated the constructive effects of security priming on the regulation of cognition and emotion. For example, security priming reduces biases in relevant information processing and results in more positive affect (Rowe and Carnelley, 2003). It also leads to increases in cognitive openness (Mikulincer and Arad, 1999), creative problem solving (Mikulincer et al., 2011), as well as state authenticity (Gillath et al., 2010). Its effects are demonstrably distinct from merely priming positive mood (Mikulincer et al., 2001b; Gillath et al., 2010).

Mikulincer et al. (2001b) identified that activating supportive representations of attachment figures as well as warm experiences of interaction can effectively induce this sense of attachment security. Such a state can produce feelings of safety and protection, which decrease defensive self-enhancing motivations, thus reducing distorted perceptions of reality (Arndt et al., 2002). It can also nurture a positive self-image, thus promoting flexible and positive interpretations of life events (Mikulincer and Florian, 2001; Mikulincer and Shaver, 2007; for review, Gillath et al., 2008). Therefore, security priming, by diminishing the attributional bias of HOES, and nourishing the poor self-image of HPES, may attenuate the maladaptive character of both attributional styles.

In the present study, we propose two hypotheses. First, compared to controls, security-primed individuals with HOES will show a less excessively self-serving attributional pattern. Second, in comparison to controls, security-primed individuals with HPES will exhibit a less depressogenic attributional pattern.

## Materials and Methods

### Participants

Three-hundred undergraduates from several universities in Beijing completed Part A of the Attributional Style Questionnaire (ASQ-A). Individuals who scored in the top 20% on overall optimistic attribution formed the HOES group, while those in the bottom 20% formed the HPES group (Chen, 2013). Of those selected, six individuals dropped out. 114 undergraduates (45 females) participated in the main experiment, their ages ranging from 17 to 27 (*M* = 20.26, *SD* = 1.96).

### Material

#### Attributional Style Questionnaire (ASQ)

To control for practice effects, we equally divided the 36 self-report items of the ASQ (Peterson et al., 1982) into A and B versions to measure individuals’ explanatory style before and after security priming. Both A and B versions contain 18 items based on 3 positive and 3 negative events. Each event has three items rated (using a 7-point Likert-type scale) on three dimensions of attribution: internal-external, global-specific, and stable-unstable. The resulting six dimensional scores – the three dimensions applied to both positive and negative events – were: (1) Internal-External Attribution toward Negative Events (IN), (2) Global-Specific Attribution toward Negative Events (GN), (3) Stable-Unstable Attribution toward Negative Events (SN), (4) Internal-External Attribution toward Positive Events (IP), (5) Global-Specific Attribution toward Positive Events (GP), and (6) Stable-Unstable Attribution toward Positive Events (SP). Then, we calculated average scores toward positive events (CP; Cronbach’s α = 0.691 in pre-priming and 0.723 in post-priming), negative events (CN; Cronbach’s α = 0.737 in pre-priming and 0.693 in post-priming), and overall scores of explanatory style (CPCN). A higher CPCN indicates a more optimistic explanatory style (Schulman et al., 1989). This questionnaire has been validated in a Chinese sample (Wang and Zhang, 2006), and showed good internal consistencies in the present study.

#### Security Priming

We used security-activation stories (Mikulincer et al., 2001a) to activate the mental representation of supportive attachment figures. The stories depict situations where someone (who is of the same gender as the participant) receives help from one of their significant others. For example, a man returns home and finds his keys missing. He asks for help from his mother. His mother stops her work and returns home immediately to assist. Additionally, as a manipulation check, we asked participants to rate how much they agreed (on a 7-point Likert-type scale) with feeling “safe,” “warm,” “supported,” and “wanting to give a hug.” A higher score demonstrated a higher degree of security activation. These four items showed excellent internal consistency in the present study (Cronbach’s α = 0.942).

### Procedure

The present study was approved by Beijing Normal University’s Institutional Review Board and had two stages. In the first stage, 300 undergraduates in Beijing were recruited to complete the ASQ-A. As aforementioned, HOES and HPES groups were formed based on their very high and very low overall scores of explanatory style, respectively. Two weeks later, these two groups were invited to take part in the second stage of the study. The 114 participants signed the experiment consent form and were randomly assigned into either a security priming condition or a control condition. Participants in the priming condition read the security-activation stories, while those in the control condition read a washing machine operating manual, a standard control procedure (Mikulincer et al., 2001a). Afterwards, participants from both groups completed the manipulation check as well as the ASQ-B as post-priming measures. Finally they were debriefing and awarded 15 RMB (The official currency of China).

## Results

### Preliminary Analysis

An independent *t*-test demonstrated a significant difference in CPCN before priming between the HOES (*M* = 4.88, *SD* = 1.78) and HPES groups (*M* = 0.04, *SD* = 1.56), *t*(112) = 15.47, *p* < 0.001, *d* = 2.924, and no significant difference prior to priming between the security (*M* = 2.58, *SD* = 2.52) and control conditions (*M* = 2.33, *SD* = 3.36; *p* = 0.657). Detailed descriptive statistics after security priming as measured by ASQ-B are shown on **Table 1**.

**Table 1:** Descriptive statistics among study variables.

	HOES (*n* = 57)		HPES (*n* = 57)	
	Priming group (*n* = 29)	Control group (*n* = 28)	Priming group (*n* = 29)	Control group (*n* = 28)
	*M* (*SD*)	*M* (*SD*)	*M* (*SD*)	*M* (*SD*)
IP	4.86 (0.80)	5.34 (0.53)	5.10 (0.54)	4.67 (0.76)
SP	5.61 (0.86)	5.86 (0.58)	5.45 (0.57)	5.46 (0.90)
GP	4.95 (1.15)	5.18 (0.93)	5.35 (0.91)	5.35 (0.80)
IN	4.69 (0.89)	4.85 (0.79)	4.54 (0.89)	4.83 (0.69)
SN	4.68 (1.16)	4.39 (1.11)	5.07 (0.92)	5.15 (0.82)
GN	4.46 (1.22)	4.31 (1.16)	4.99 (1.05)	5.13 (0.74)
CP	15.43 (2.19)	16.38 (1.55)	15.91 (1.58)	15.48 (2.03)
CN	13.83 (2.01)	13.55 (2.18)	14.60 (2.25)	15.12 (1.79)
CPCN	1.60 (1.97)	2.83 (2.12)	1.31 (1.85)	0.35 (1.58)

*IP, internal-external to positive events; SP, stable-unstable to positive events; GP, global-specific to positive events; IN, internal-external to negative events; SN, stable-unstable to negative events; GN, global-specific to negative events; CP, average score to positive events; CN, average score to negative events; CPCN, overall score of explanatory style.*

### Security Priming Check

An independent *t*-test showed that participants in the security priming condition (*M* = 5.35, *SD* = 1.38) had a significantly higher sense of security than participants in the control condition (*M* = 2.76, *SD* = 1.36), *t*(112) = 10.09, *p* < 0.001 and *d* = 1.906. Thus, we successfully activated their sense of attachment security.

### Effects of Security Priming on Explanatory Styles

**FIGURE 1:**
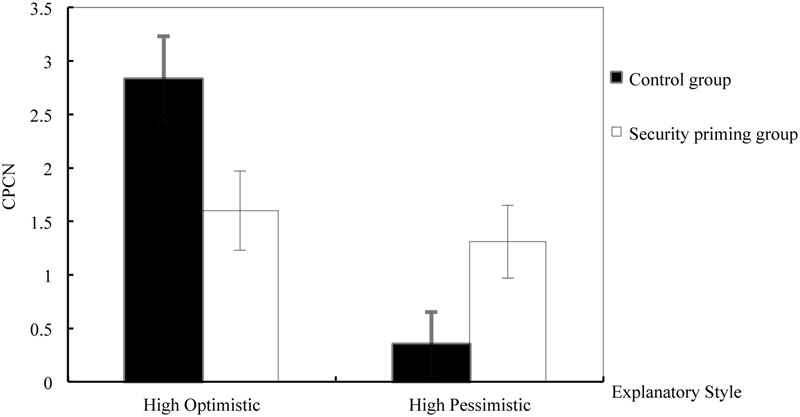
**Effects of security priming on the overall level of explanatory styles (CPCN)**.

A Priming (security priming vs. control conditions) × Explanatory Style (HOES vs. HPES groups) two-way ANOVA on CPCN after priming revealed that the main effect for Priming was not significant (*F* < 1), whereas the main effect for Explanatory Style was highly significant, *F*(1,110) = 15.19, *p* < 0.001, $\eta_{p}^{2}$ = 0.121. That is, participants in the HOES group showed a significantly higher level of optimistic attribution when compared to the HPES group. The interaction of Priming and Explanatory Style was also significant, *F*(1,110) = 9.53, *p* = 0.003, $\eta_{p}^{2}$ = 0.080. Simple effect analysis demonstrated that participants with HOES showed significantly lower optimistic attributions in the security priming condition than in the control condition, *F*(1,110) = 6.07, *p* = 0.015, $\eta_{p}^{2}$ = 0.052. As for participants with HPES, there was a marginally significant increase in CPCN in the security priming condition, *F*(1,110) = 3.61, *p* = 0.060, $\eta_{p}^{2}$ = 0.032. Thus, while priming participants with attachment security decreased optimistic attributions for the HOES group, it increased optimistic attribution among participants with HPES. Moreover, while the HOES and HPES groups differed significantly on optimistic attribution in the control condition, *F*(1,110) = 23.97, *p* < 0.001, $\eta_{p}^{2}$ = 0.179, this discrepancy disappeared in the security priming condition (*p* = 0.564). See **Figure 1**.

**FIGURE 2:**
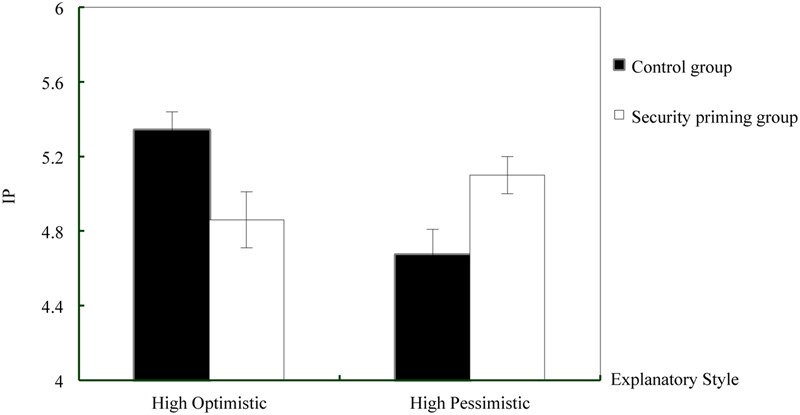
**Effects of security priming on the dimension of internal-external to positive events (IP)**.

Identical analyses were used to investigate the effects of security priming on the six dimensions of explanatory style. We found a significant interaction with Priming and Explanatory Style in the IP dimension, *F*(1,110) = 13.44, *p* < 0.001, $\eta_{p}^{2}$ = 0.109, but no significant interactions on the other dimensions (*p*s > 0.331). Simple effect analysis revealed that, compared to the control condition, security-primed participants with HOES attributed positive events more externally *F*(1,110) = 7.41, *p* = 0.008, $\eta_{p}^{2}$ = 0.063. In contrast, for participants with HPES, security priming resulted in more internal attributions of success *F*(1,110) = 6.06, *p* = 0.015, $\eta_{p}^{2}$ = 0.052. Therefore, security priming significantly increased internal attributions of success for the HPES group, while significantly reducing this for the HOES group. Moreover, while HOES participants internally attributed positive events significantly more than HPES participants in the control condition, *F*(1,110) = 14.37, *p* < 0.001, $\eta_{p}^{2}$ = 0.116, this difference disappeared in the security priming condition (*p* = 0.173). See **Figure 2**.

## Discussion

The present study was the first to explore the effects of security priming on the maladaptive patterns of HOES and HPES. Consistent with our hypotheses, activating mental representations of attachment security significantly reduced the overly self-serving attributional pattern of HOES individuals and marginally improved the depressogenic attributional patterns of HPES individuals. Although we would caution in concluding that HOES and HPES are thus rooted in attachment insecurity, the present study empirically supports the efficacy of security priming on moderating both HOES and HPES.

For individuals with HOES, we find this balancing effect in their decrease of self-serving attributional bias. As introduced above, HOES is closely associated with this bias, which defensively attributes positive outcomes internally and negative outcomes externally, in order to prevent negative information from entering the self-concept (e.g., Campbell and Sedikides, 1999). This attributional pattern is characteristic of the defensive self-enhancement tendencies of avoidant-attached individuals (Kogot, 2002, unpublished). In the present study, however, we found that security primed HOES individuals attributed successful events more externally, which contrasts with their attributional bias. This result suggests that priming loving representations of significant others can render their defensive self-enhancing maneuvers less necessary. This lends truth to the idea that both chronic and contextual activation of attachment security constitutes a primary source of self-protection and authentic self-worth, both of which attenuate defensive self-enhancing motivation (Mikulincer and Shaver, 2007).

As for individuals with HPES, this adaptive effect is expressed in their increased level of optimistic attribution. As a result of their negative self-image, HPES individuals exhibit an explanatory pattern characterized by helplessness and passivity (Peterson et al., 1992). They have lower expectations, are more concerned about potential failures, and are less efficacious (Eronen et al., 1998). This explanatory style is characteristic of insecurely attached anxious individuals. Such individuals regard helplessness and vulnerability as a way of evoking others’ help and care, and thus tend to ascribe success externally out of a defensive, self-deprecating strategy (Bartholomew and Horowitz, 1991; Mikulincer and Florian, 2001). However, by activating supportive representations of attachment security in a context of need, we find that security-primed individuals with HPES show a higher level of optimistic attribution. Specifically, these individuals made significantly more internal attributions for successful events. As suggested by Mikulincer and Shaver (2005), during the course of security priming, individuals experience being seen as special and protected by a responsive attachment figure; this in turn allows them to perceive themselves as competent, valuable, and efficacious. Hence, warmly activating significant others and recalling experiences of security reduces HPES individuals’ helplessness and obviates this defensive and self-defeating attributional strategy.

Furthermore, we suggest that a possible mechanism for the salubrious effect of security priming on HOES and HPES may be the improvement of these individuals’ self-representation. According to attachment theory, attachment insecurities are associated with a series of maladaptive strategies in appraisal and interpretation (for review, Mikulincer and Shaver, 2012). These maladaptive strategies are mediated by dysfunctional beliefs, which also distort their self-representation. While a strategy for an anxiously attached person may involve a self-defeating process, that of an avoidant person may involve self-enhancing defenses; both, however, destructively bias their self-representations (Shaver and Mikulincer, 2011). Therefore, we believe that activating attachment security attenuates these maladaptive attributional patterns by improving dysfunctional self-beliefs. Specifically, strengthening the accessibility of supportive representations of attachment security nurtures a more constructive self-representation where an attachment figure is available and responsive in times of need. Once these individuals feel more secure, they no longer need to depend overly on others’ approval or to exaggerate their strengths.

In summary, our findings indicate that contextually strengthening the accessibility of mental representations of attachment security not only attenuates the defensively self-enhancing attributions of individuals with HOES, but also improves the depressogenic attributions of individuals with HPES. This illustrates how security-priming method can help people explain life events more adaptively and realistically, instead of distorting or inflating their attributions. Additionally, the present study provides a novel direction in understanding the relationship between attachment insecurity and dysfunctional explanatory patterns. While previous studies have described the dysfunctional explanatory patterns of insecure attached individuals (e.g., Webster, 2002), the present study is the first to examine the effect of activating attachment security on these individuals. This effect tentatively suggests a causal relationship between attachment insecurity and dysfunctional attributions.

Based on the limits of this study, we propose four areas of future research. First, we did not measure possible mechanisms underlying this attenuating process, including self-representation. Further study can investigate such mechanisms. Second, further work should consider the potential moderating role of individual differences on this attenuating process. These include differences in trait attachment styles as well as with clinical populations. For example, depression is associated with a depressive attributional style (Mezulis et al., 2004), and attention-deficit/hyperactivity disorder (ADHD) and pathological narcissism are associated with grandiose self-perception and self-serving attributional bias (Hoza et al., 2000;Dickinson and Pincus, 2003). Third, further study can examine HOES to more precisely distinguish the subset of individuals whose optimistic explanatory style is excessive and unwarranted from those who adopt a moderate and adaptive optimistic explanatory style. Moreover, to further validate the finding of the present study, further work can adopt different approaches to measure explanatory styles, such as video coding methods.

## Conclusion

The present study conceptually differentiated HOES from optimistic explanatory style in general, and was the first to experimentally test security priming on dysfunctional explanatory patterns. While HOES and HPES consist of differing defensive strategies, we found that security priming attenuated both these dysfunctional patterns. Activating supportive representations of loved ones led HOES individuals to attribute less defensively, while helping HPES individuals to attribute more optimistically. While mechanisms and moderating factors remain to be explored, this study provides a nascent understanding toward therapeutic applications where a realistic understanding of events is valued.

## Author Contributions

YD, MY, and YL designed and executed the study. YD, HC, and XL reviewed articles. YD, PZ, and XS analyzed the data. YD, HC, and XZ wrote the paper. All authors discussed the results and commented on the manuscript.

## Conflict of Interest Statement

The authors declare that the research was conducted in the absence of any commercial or financial relationships that could be construed as a potential conflict of interest.

The reviewer AM and handling Editor declared their shared affiliation, and the handling Editor states that the process nevertheless met the standards of a fair and objective review.
